# Long-term consequences of food insecurity among Ebola virus disease-affected households after the 2013–2016 epidemic in rural communities of Kono District, Sierra Leone: A qualitative study

**DOI:** 10.1371/journal.pgph.0000770

**Published:** 2022-10-31

**Authors:** Manuella L. Djomaleu, Abu B. Rogers, M. Bailor Barrie, George W. Rutherford, Sheri D. Weiser, J. Daniel Kelly

**Affiliations:** 1 School of Medicine, University of California, San Francisco, San Francisco, California, United States of America; 2 School of Medicine, Stanford University, Stanford, California, United States of America; 3 Institute for Global Health Sciences, University of California, San Francisco, San Francisco, California, United States of America; 4 Partners In Health, Freetown, Sierra Leone; 5 Department of Epidemiology and Biostatistics, University of California, San Francisco, San Francisco, California, United States of America; 6 Division of HIV, Infectious Disease, and Global Medicine, Department of Medicine, University of California, San Francisco, San Francisco, California, United States of America; 7 F.I. Proctor Foundation, University of California, San Francisco, San Francisco, California, United States of America

## Abstract

The 2013–2016 Ebola virus disease (EVD) epidemic caused food insecurity during and immediately following local outbreaks in Sierra Leone, but longer-term effects are less well described, particularly among households with no EVD survivors. We conducted a qualitative sub-study in July 2018 in Kono District, Sierra Leone to understand the impact of food insecurity on EVD-affected households. Using data from a community-based cohort, we compiled a list of all households, within the sampled communities in Kono District, that had at least one EVD case during the epidemic. We used purposive sampling to recruit 30 households, inclusive of 10 households with no EVD survivors, to participate in the study. The research team conducted open-ended, semi-structured interviews with the head of each household. All 30 interviews were transcribed, translated, and analyzed using comparative content analysis consistent with a grounded theory approach. Most household members were facing persistent food insecurity as direct or indirect consequences of the EVD epidemic, regardless of whether they did or did not live with EVD survivors. Three major themes emerged as drivers and/or mitigators of EVD-related food insecurity. Financial instability and physical health complications were drivers of food insecurity in the population, whereas support provided by NGOs or governmental agencies was observed as a mitigator and driver of food insecurity after its removal. Among the EVD-households reporting long-term support through jobs and educational opportunities, there was sustained mitigation of food insecurity. EVD-affected households with and without survivors continue to face food insecurity three years after the EVD epidemic. Provision of support was a mitigator of food insecurity in the short term, but its removal was a driver of food insecurity in the longer term, suggesting the need for longer-term transitional support in affected households.

## Introduction

The 2013–2016 Ebola virus disease (EVD) epidemic caused widespread health system and socioeconomic disruptions in Sierra Leone, Guinea, and Liberia, resulting in food insecurity. The failure of governments to mount an adequate response early in the epidemic promoted distrust in the healthcare system because of the potential for EVD transmission within facilities [1, 2]. In the absence of a functioning health system, health providers could not assess food insecurity, which has been associated with poor mental health and worse clinical outcomes, including Human Immunodeficiency Virus (HIV) and EVD [3–7]. Widespread socioeconomic disruption in the affected countries compounded issues drastically increasing food insecurity because of increased unemployment, business closures, and other challenges [8–12].

The current EVD literature largely prioritizes containment strategies during an outbreak and disease transmission and outcomes, with few studies focused on the long-term socioeconomic impacts of outbreaks [13, 14]. The few studies that have been conducted in the Democratic Republic of Congo (DRC) and Liberia on the long-term socioeconomic impact of EVD are more broad focusing on agricultural production, lost wages, and economic stability [9, 15–17]. We chose to focus on food insecurity due to its high prevalence in Sierra Leone prior to the epidemic, and because food insecurity is a specific marker of poverty that can be widely assessed across various groups [18]. The other markers of socioeconomic stability, such as occupation, education, and income levels, vary among communities so these were not used as the primary measure of the socioeconomic impact of Ebola; however, they were still incorporated into the study.

Food insecurity, defined as an inability to obtain food in a socially acceptable manner, impacted approximately 45% of Sierra Leone’s population of 7.3 million prior to the epidemic [18, 19]. Policies to reduce the spread of EVD such as market closures and trade restrictions further pushed an additional 750,000 people into food insecurity, including farmers, unskilled workers, fishermen and rural community members [9, 20–22]. Given the 2018–2020 EVD outbreak in the DRC and the 2020 COVID-19 pandemic, populations in West and Central Africa were highly susceptible to the economic effects of viral outbreaks, with food insecurity as a potential downstream effect of such health crises [16, 23, 24]. The few studies on the long-term socioeconomic effects of an EVD outbreak on food security largely focused on survivors [9], leaving out the social well-being of the entire household unit including households without EVD survivors [25].

EVD affected the social relationships of infected individuals, particularly their household unit. Out of 1.2 million households in Sierra Leone, EVD affected 6,951 households across the country, with EVD clinical outcomes ranging from one or more household members dying, all household members surviving the infection, and some households experiencing a combination of the two [26]. In a setting where EVD case fatality rates were high (approx. 50%) [27], many affected households lost family members who were critical to their financial stability [25]. When a working parent died, the loss of income and poverty levels left children vulnerable to food insecurity.

The outbreak affected the socioeconomic stability of households in various ways. Firstly, some households with EVD survivors experienced exacerbating effects of financial instability because survivors may have suffered physical sequelae resulting in an inability to work [28–32]. Although EVD survivors received a range of support services (ie. one-time food provision, employment, education) from governmental or nongovernmental organization (NGOs) following infection for at least three months [25, 33], the heterogenous distribution of support led to differences in survivors’ wellbeing in the long term. The only survivors who were food secure two years after the epidemic were those who had received at least six months of employment or education [25]. Survivors who only received food assistance without transition to independence may have suffered from food insecurity once NGOs transitioned their programs. Secondly, public health mandates resulted in substantial socioeconomic consequences for the household unit. For example, quarantined houses required decontamination, which sometimes meant destroying all household belongings [5, 9, 34]. As a result, some households lost their stored food supplies. Lastly, some households were unable to harvest their crops in a timely manner because of the quarantine period, which resulted in food insecurity [13].

## Methods

### Ethics

The University of California, San Francisco (UCSF) Institutional Review Board and the Sierra Leone Ethics and Scientific Review Committee reviewed and approved the parent study and this qualitative study (Ethics Approval #:15–17827). Participants provided written consent for the parent study and verbal consent for the interview.

### Overall study design and setting

After the EVD epidemic in West Africa, we recruited a cohort to investigate the social and serological epidemiology of EVD in several rural communities in Kono District, Sierra Leone. Within this cohort, we conducted a qualitative sub-study of EVD-affected households with the objective of determining drivers of food insecurity in these households three years after the end of the epidemic. Given that households in which there were no EVD survivors (all decedents) were generally not part of assistance programs (no benefits), we intentionally sampled EVD-affected households with and without survivors to describe the potential range of experiences with food insecurity.

We conducted this qualitative sub-study in July 2018 using semi-structured interviews in Kono District, Sierra Leone. Kono District, one of twelve districts in Sierra Leone, is a semi-urban community with a population of 500,000 and home to Sierra Leone’s largest diamond deposits [35, 36]. Most people in Kono District relied on daily earnings from mining, farming, or selling farm produce as their primary source of income [36]. Prior to the outbreak, the region was the epicenter of the civil war in the 1990s and early 2000s due to its mineral deposits [37, 38]. During the EVD outbreak, Kono was one of the last districts in Sierra Leone to experience a surge in EVD cases, with transmission chains in Joe Town, Ngo, Ndogboya and Bumpe [39]. When these communities experienced local EVD outbreaks in late 2014 and early 2015, the Ebola response was stronger than in early 2014, and support services were rapidly available to EVD survivors. After the epidemic, Kono received an infusion of resources from the World Bank and NGOs leading to road development and health systems strengthening [40, 41].

### Study population

The study population of our parent cohort included quarantined households with at least one reported EVD case (survivor and/or decedent) who lived in Joe Town, Ngo, Ndogboya and Bumpe during the local EVD outbreak in 2014. EVD cases were identified through the district’s Viral Hemorrhagic Fever (VHF) database and confirmed through interviews with community leaders, the Ebola Survivor Association and healthcare workers. Quarantined households were identified through Kono District Ebola Response Center (DERC) and confirmed through interviews with community leaders and EVD survivors. The quarantined households included all the reported EVD cases. In households with EVD survivors, we identified their close contacts, confirmed their exposure history and enrolled them in our study [39].

A member of the research team visited each participant’s household. During this visit, participants completed an epidemiological survey, blood draw, and participated in an open-ended interview (focused on story of acute EVD within household and transmission events). Within this parent cohort, there were 497 participants (76 reported EVD cases, 421 contact-participants). Nearly half (48%) of participants were between ages 20 and 49. The majority were male (58%). In both instances, less than one-third completed primary education (28%) and secondary education (31%). A high proportion (58%) were farmers or miners. This study population was food insecure prior to the EVD epidemic [5].

We used quarantined household data from our community-based cohort to guide a purposive sampling strategy to select 30 EVD-affected households out of the 497 households in the parent cohort with the goal of having equal representation from households with no EVD survivors, households with only EVD survivors, and mixed households with EVD survivors and deaths. The head of each eligible household was invited to participate in a semi-structured interview on their lived experiences as they pertained to food security after the Ebola epidemic.

### Data collection

A trilingual member of the research team (M.B.B.) conducted all interviews in Krio or Temne, which lasted thirty to four-five minutes. M.B.B is a male Sierra Leonean from Makeni who has worked in Kono District since 2006. During the outbreak, M.B.B worked in Kono District with NGOs to establish a response and continues to work there at the time of this study. All interviews were conducted in the participants’ homes and audio-recorded for data collection. Participants did not receive access to the audio recordings or interview transcripts. Demographic data were collected to confirm the identity of the participants, as well as data describing household membership before and after the epidemic. Using a grounded-theory approach [42], we asked open-ended questions and probed for relevant dimensions of food security (food quality, food safety, procuring food in socially unacceptable ways, anxiety about food access and availability). Interviews lasted between thirty to forty-five minutes. We asked participants to describe their experience with food before, during, or after the epidemic; the period after the epidemic was further divided into two parts: the period immediately after the EVD epidemic in their household and the time of the study (2018).

### Analysis

All interviews were translated into English by A.B.R. (fully trilingual) and imported into Dedoose, a web-based program to organize and manage qualitative data (Dedoose 9.0.17, Sociocultural Research Consultants, Los Angeles, CA). First, using a deductive approach, where predetermined codes are applied to the data, two investigators (M.L.D., A.B.R.) organized the data around household type (presence or absence of EVD survivors) and short and long term food stability [43]. Next, the two investigators (M.L.D., A.B.R) independently developed a thematic coding framework based on the conceptual framework of food insecurity (ecological, economic, social factors) and identified new sub-themes and codes consistent with an inductive approach to qualitative analysis [43]. The two investigators discussed all codes and thematic classifications with the lead researcher J.D.K (an Ebola physician researcher who worked in the communities before, during, and after the EVD epidemic) for consensus or resolution of discrepancies. This process was repeated until saturation was reached, and no new codes emerged from the data. For each major theme, we identified a series of excerpts and present them in the results section with participant information de-identified to protect confidentiality.

Participants did not provide feedback on the findings.

### Inclusivity in global research

Additional information regarding the ethical, cultural, and scientific considerations specific to inclusivity in global research is included in the S1 Checklist.

## Results

We interviewed the head of each household, for a total of 30 interviews. Of the 30 households, 64% had ten or fewer household members while 36% had more than ten members. The mean age of the interviewed participants was 41 years, similar to the mean age of the heads of their households in the parent study. Half (50%) were men; another half (47%) were EVD survivors. All participants interviewed were over the age of 18. Of the EVD survivors who could still work, 50% relied on agriculture as a source of income and food. Almost all interviews were completed in Krio, the primary language of the participants.

Household structure remained largely heterogenous within our cohort. After the epidemic, many families sent their children to extended relatives in other towns because they could no longer take care of them (due to reduced income), and conversely extended family members often moved into households to take care of the children of deceased parents. Both situations resulted in fragmented households and families.

Prior to EVD, participants were able to procure two meals a day, which is still less than the recommended three. Most participants described worsening food insecurity after the epidemic because they had to reduce frequency of meal consumption, quantity and or quality. In some households, food insecurity from Ebola reduced the number of daily meals from two to one meal per day. In other households, participants described worsening food insecurity based on changes in food quality in which they ate less meat, which is considered to be more expensive. We identified three major themes as drivers and/or mitigators of food insecurity: (1) financial instability, (2) health complications and (3) NGO and governmental support. Financial instability and health complications emerged as drivers of food insecurity; meanwhile NGOs or governmental agencies emerged as both a mitigator and driver of food insecurity. Below we elaborate on these major themes. We also describe the role of religion and community as coping mechanisms for households experiencing food insecurity.

### Financial instability

Many households relied on agriculture or income through a small business to gain financial resources to buy food. Certain households faced food insecurity when their business were destroyed during quarantine measures and they could not recover their losses. Others faced food insecurity when either one or both breadwinners (often parents or oldest members in the housing unit) died due to EVD. In both scenarios, households suffered a reduction in income, leading to financial instability and eventually food insecurity. We identified loss of primary business and death of the breadwinner as drivers of financial instability and describe them below.

#### Loss of primary business as a direct barrier to income generation.

For households’ whose primary income source was trading, the public health response directly caused financial instability through containment and quarantine. The containment strategy required decontamination, accomplished through incineration of all household’s belongings, including business goods. A 36-year-old woman from a survivor-only household said:

When I used to do business, I kept my money under my mattress after selling items. When I got infected, I was sent to Kenema for treatment, [but] before I came back, they took out my mattress and burned it including the money I hid in it with all my other belongings.

The forty-day quarantine prevented residents from operating their business. Most residents in Kono District rely on the sale of their farm harvests to procure other food items. As a result, harvested produce spoiled during quarantine and could not be sold. A 35-year-old woman says:

Immediately after Ebola, things were hard because when they quarantined us, we lost our business. All the produce, like peppers, we bought to sell went bad because they did not allow us to leave the house.

Households that lost critical business supplies faced long-term financial consequences. Since most households did not have savings, their businesses could not recover from the epidemic.

#### Loss of breadwinners as an indirect barrier to income generation.

Participants in households with EVD deaths described challenges related to food quantity and quality. Many households had a primary breadwinner, who was responsible for financially supporting the household, and secondary breadwinners who contributed to the household income in various forms, mostly through farming. Therefore, death of breadwinner(s) due to EVD emerged as a driver of food insecurity. A 20-year-old woman from a household without survivors says:

Well, before the Ebola we worked hard. Other family members [the parents who died] worked hard so that the children will have the things they need. We ate three times a day, but since my parents died from Ebola, securing a consistent source of income for food has been harder. Things have been strenuous because both died.

When only one breadwinner died, some households still ate two meals a day. However, they were unable to eat a balanced diet. A 24-year-old woman says:

When my husband was alive, the food quality was much better… I ate meat; now I do not eat meat. The quality is not good because I am the only one … going to the farm to work to fend for my family.

In certain households where the primary breadwinner died, another relative outside the household provided financial support; however, this did not always lead to food security. A 65-year-old woman says:

*Things are hard, we do not have much. A different person takes care of us now, so it* [food quality] *is not the same as before. Things were better for us before the Ebola because my child took care of us, but he died during the Ebola. Now, someone else* [extended family member] *takes care of me so I have to manage whatever little he can provide.*

### Long-term sequelae amongst EVD survivors

Our study population primarily engaged in farming and mining for income, both physically intensive occupations. EVD survivors described ongoing physical sequelae, including generalized body pain, weakness, and vision problems, limiting their functional ability to engage in physical labor. Among EVD survivors, EVD-related disability emerged as a driver of food insecurity.

#### Post-EVD sequelae as a direct barrier to farming and mining, resulting in food insecurity.

Survivors were unable to farm as frequently as pre-EVD era due to body weakness and pain. A 59-year-old farmer says:

Well, I still eat just not sufficient. My body is different now. In the past, I was able to do hard work. However, now I can’t. My whole body hurts if I do hard work after I got sick from Ebola.

Other survivors who were the primary breadwinners could not return to faming due to sequalae, resulting in a change to household structure. A 62-year-old male farmer says:

I sent two of my children to Freetown because I do not have a job to feed them… Well, it is because I got sick. I am no longer able to do things again because my body hurts and is weak-…My eyes also hurt. They took a look at my eyes a while ago, and they said that I have jaege (cataract).

The remainder of this participant’s children became the primary breadwinners for the family, given he could no longer work. As a result, the daily meals of the household were reduced from two to one meal.

Right now, my three children go get wood, and they sell it for 5,000 leones. That is how we survive. I am scared. When they go out, I have to follow them because the country is not very safe…I do not like it at all. I feel bad because this is not something we used to do. I was able to get their food and provide for them.

#### Lack of consistent follow-up on management of sequelae.

Limited access to healthcare services also exacerbated the effects of post-EVD sequelae. Some participants were unable to get medications or follow up care, which further impeded their ability to work and bring food for their family. A 59-year-old man says:

But since I got Ebola, it has been hard. I can no longer get medicine from where I used to get it because they said that Ebola is over now, so they no longer provide it to us. When I earn some money, I buy some medicine that lasts two or three days. Once the drug is done, the effect of the drug is over.

This participant primarily relied on agriculture for food procurement, however EVD-related sequalae has made it difficult for him to work.

Well now my body is different. In the past, I was able to do hard work [referring to agriculture]. However, now I can’t. After Ebola, my whole body hurts if I do hard work.

As a result, the lack of medical support to address his pain, further contributed to his food insecurity.

#### The exacerbating effects of physical sequelae among EVD survivors who were primary breadwinners.

Households where breadwinners experienced sequelae were more likely to experience food insecurity than households where no one experienced sequelae or where household members other than the breadwinner experienced sequelae.

A 55-year-old male survivor who was the primary breadwinner for his family says:

Yes, it was so much better [before Ebola]. I was able to fend for something to eat. Now, I don’t have chance (means) and I don’t have anything to eat in the house. My friends help me from time to time… I was able to work before Ebola. But now after I got sick, I am not able to work. It is difficult for me… Now that God arrested my ability (sick). In the past I would go to the bush (farm) and whatever we got I was able to use it to survive.

### NGO and governmental support

Households with EVD survivors received support from NGOs and governmental organizations through direct food provision, educational and employment opportunities for at least three months after their EVD-infection. During this time, they reported a high level of food quality and consumption. Three years afterwards, most NGOs had transitioned out of the area with varying levels of long-term support. Major sub-themes emerged as consequences of NGO and governmental support: (a) provision of short-term support, (b) lack of short-term support, (c) withdrawal of support with and (c) without transitions to independence. Though the provision of short-term support and long-term employment were mitigators of food insecurity, the withdrawal of support from these household became a driver of food insecurity, particularly among those lacking long-term employment opportunities.

#### Provision of short-term support.

Since households with EVD survivors were the focus of aid in the international NGO response, they were able to receive socioeconomic resources that mitigated food insecurity. These households described themselves as more food secure with a better quality of life. A 62-year-old man says:

After the Ebola, [NGO] have been helping me with the kids. They paid for their school fees. They also supplied school bags and uniforms. They did not give us actual food, but they gave us money. For each child, they gave us 60,000 leones with four packets of water.

In some cases, the support provided a higher quality of life than households experienced before the epidemic. A 35-year-old woman says:

When Ebola was over, they brought supplies for us. Things were not strenuous then. The time they provided supplies for us was better than before the Ebola.

#### Lack of short-term support in households with no EVD survivors.

Households with no EVD survivors were not prioritized in the humanitarian response. Some of these households only received support during the Ebola quarantine period. Subsequently, these households experienced a high burden of food insecurity, especially in households where the primary breadwinner died. A 35-year-old woman whole lost her sister, the primary breadwinner says:

No, they [NGO] only provided us with supplies during the quarantine period. After quarantine was over, they did not give us any other supplies.

She reported her family ate two meals a day before the Ebola, but now her family struggled to secure one meal a day.

… We only eat when we have food. Right now, I am unemployed… you still have to feed your family even when things are difficult.

#### Withdrawal of short-term support without a transition to independence.

Households with survivors had access to high-quality food ate three times a day, when they received consistent nutritional support from NGOs. As many NGOs transitioned out of the area, these households lost access to food. The transition of NGO support required households to return to their reliance on internal resources, so households in which the breadwinners had died, lost business supplies, or were experiencing work-limiting sequelae struggled with food insecurity. Participants who did not receive any employment or educational opportunities from NGOs, reported being food insecure once they no longer received food supplies. A 35-year-old woman described how after NGO efforts terminated, the food quality in her household gradually declined until it was worse than pre- EVD due to the exacerbating effects of the loss of primary breadwinners.

…Since they stopped bringing us supplies, things have been strenuous, especially with the children. It has been strenuous to pay their school fees and get food to eat.… when Ebola was just over, they brought supplies for us. Things were not strenuous then. Now, it is much difficult because they stopped helping us. They have abandoned everything (us).

A relative of one of the Ebola survivors describes the changes in the food quality in his household when the NGO support ended. The household had been the victims of theft during the epidemic, consequently they were unable to restart their farm before the NGO transition. This household did not receive any resources to help rebuild the farm, nor did the family have any savings to buy new animals for the farm.

During that time [when supplies were provided by NGO], we ate twice… The time they provided food for us was better. Now we only eat once a day… Before Ebola was better because now things are not so easy. Ebola disrupted a lot of things…when I got sick and my dad went with me, people stole the animals on his farm.

While many NGOs provided food and school supplies, a few aimed to have long-term impact in the lives of EVD survivors by providing them with employment and/or educational opportunities. However, some job or educational opportunities were short-lived and ended when NGOs transitioned out of the areas and/or programs ended. For example, a 42-year-old male EVD survivor who was employed by an NGO after the Ebola epidemic explained how his job provided him with financial security.

Well, the support I meant is that after I got home from Ebola, [NGO] gave me a job. I was able to use the money I earned from the job to support my family… when I had a job, I used the salary to cover a lot of things, including the children’s school fees. I also used it to take care of the children who lost their parent to Ebola.

However, when he later lost his position, he was unable to consistently secure two meals a day.

I no longer have that job, though… They fired me and some of the other survivors. I no longer have the health or stamina to do farming. I plant okra because it only takes forty days. My health is not strong enough to do heavy farming. At least when I plant okra and corn, I am able to sell some of that to get food for me and my children…I am not always able to eat twice a day because things are difficult for us right now.

#### Sustained support of EVD survivors as a mitigator of food insecurity.

A few survivors received employment opportunities from NGOs that continued until the study time period. These participants expressed having a better quality of life. A 45-year-old male survivor says:

Not too long after returning from the treatment center, they called me at the social welfare to take care of kids and I began working there and received a salary. There was no problem getting food on a daily basis…Things are better now than before the Ebola.

Another 34-year-old male survivor received employment from an NGO. He described stable food security, punctuated by improved quality of life due to adult literacy programs that increased his socioeconomic stability.

I still eat the same things I ate before. I do not have to worry about my children’s school fees. Through [NGO], they gave us a lot of opportunity. I didn’t go to school but through the programs and adult education, I have learned a lot. I can fill forms on my own now. I can write my name and sign all because of [NGO]. I am much more knowledgeable now, so things are much better.

All participants who remained employed over the long term transitioned to independence from the provision of short-term support.

### Coping mechanisms

Our study population relied on support from NGOs to directly obtain food or obtain resources (i.e. education, job security) that improved their ability to secure food. When NGOs were unable to provide them with such support, some individuals looked to their community to obtain food or resources needed to buy food. A thirty-two-year-old male survivor says:

To be honest with you, now I have to go to my brother, who is the chief, to survive. We have to go to the swamp now. When we get back, he gives us our 10 so that we can survive.

Religion emerged as a coping mechanism to help participants process the adverse effects of food insecurity and their lived experience of EVD. Many participants discussed inconsistent access to food or work, however they remained hopeful about the future due to their faith in God. A 24-year-old woman who lost her husband, the primary breadwinner, says:

I thank God for my life and good health. My heart is content, and I am happy that my child was able to continue his education after his illness. Whatever God has planned for us for the day, we know that we will survive.

## Discussion

This is the first qualitative study to explore the long-term impact of the 2014 Ebola epidemic in Sierra Leone on food insecurity in EVD-affected households. The results from our study show most EVD-affected households dealt with worsened food insecurity three years after the epidemic. Using a social-ecological framework [43], we identified three main drivers of food insecurity in EVD-affected households: financial instability, long-term physical health sequalae, and lack of long-term support.

In our study, death of a breadwinner or loss of their resulted in major changes to the structure and support system of households and the annual income of these households. While the outbreak had a direct influence on the financial stability in many households, the systemic impact of the disease also drove our participants into financial instability. Prior to the Ebola epidemic, many of the households in our study struggled financially due to Sierra Leone’s economic situation, as the nation was still recovering from the perils of the civil war [44]. When the epidemic began, Sierra Leone was finally making great strides in economic growth, with the country’s GDP ranked amongst the fastest growing in 2014 [45]. However, during the outbreak, there was a substantial loss in productivity, and an increase in food prices [46] pushing an additional 750,000 people into food insecurity [5]. Sierra Leone lost an estimated $1.9B from the Ebola epidemic [47]. There was a decrease in revenue generated from the mining and farming sectors, which disproportionately impacted Kono district. All of these systemic and structural factors worsened the financial stability of many households. Consequentially, breadwinners of EVD-affected households, who needed new sources of income, struggled to find jobs in the declining economy. Households that lost business due to the EVD quarantine protocol were not able to recover from their financial losses. Therefore, the effects of the systemic and structural factors in combination with the major changes that were happening at the household level in EVD-affected households amplified financial instability, which made level of food insecurity in EVD-affected households more profound than non-EVD affected households. Similar to our findings that financial instability serves as a driver of food insecurity, a study conducted by Josephson and colleagues showed that the COVID-19 pandemic has resulted in food insecurity in many households in Ethiopia, Malawi, Nigeria and Uganda because of the pandemic’s direct impact on financial instability [16, 23, 24].

In many EVD-affected households, survivors experienced debilitating sequelae that prevented employment involving physical labor (i.e., mining, farming). Because many of the households in our cohort and in Kono district relied on blue collar jobs for their income, the debilitating post-EVD sequalae had a direct impact on food security. These households also experienced a loss in productivity and reduction in household income. Similar to financial instability, systemic and structural factors also influenced the role of long term sequalae on food insecurity. The poor healthcare infrastructure in Kono district and other parts of Sierra Leone made it difficult for survivors to access care. Prior to Ebola, Sierra Leone had a shortage of healthcare providers. The epidemic made this shortage worse as the country lost 7% of its healthcare workers [48]. As a result, the healthcare system was not prepared to handle the novel medical needs of EVD survivors who presented with post-EVD sequalae. Many of the participants in our study reported that they struggled to make ends meets as their health deteriorated, which further exacerbated financial instability and food insecurity in their households. Additionally, because of the financial instability inflicted on the households by Ebola, many of the survivors could not afford to seek care from profit, private healthcare institutions.

During and immediately after the epidemic in Sierra Leone, there was a national government effort to provide support through a Comprehensive Programme for Ebola Survivors (CPES) [49]. These services included access to healthcare through the Free Health Care Initiative program, access to free medications, and transportation support for referrals to sub-specialty care. NGOs provided food and additional support to households with EVD survivors [33], enabling them to meet their dietary needs during that time period. While the provision of services was important to the health and well-being of EVD survivors, they did not adequately target the pathways of food insecurity identified in our study (e.g., financial instability, work-related disability) because of their breadth, duration, and scope. In terms of breadth, CPES and other programs were focused on the EVD survivor and not the household unit. Most households consisted of a large, extended family with one or two primary breadwinners, highlighting the point that even though the Ebola virus infects individuals, the impact of the disease is felt by everyone in the household unit.

In terms of duration and scope, most EVD survivors received NGO support for less than a year, which was further limited in scope of health services. Few survivors received employment opportunities. Studies have shown that when EVD-survivors received job security, educational opportunities and monetary support for at least six months, these survivors experienced a better quality of life [25, 50–52]. Depending on how long support was continued by organizations in our study, some households with EVD survivors remained food secure after support was removed. These findings extend evidence that when survivors received comprehensive, long-enough socioeconomic support through job security and education, it allowed households to increase their income, eventually leading to long-term food security and improved social wellbeing [25]. While long-term socioeconomic support is critical to ensuring affected communities can be financially secure once NGOs transition their support out of the area, these investments often take time before individuals can accrue the benefits of such programs [53,54]. We found that for most NGOs, the removal of support was too early to sustain the gains needed for long-term food security and stability, pointing to the need for more comprehensive approaches (e.g., cash transfers, continuous food provision) in supporting households directly impacted by EVD, not only survivors, over a longer period.

The provision of support was not equal among households. Households that only had EVD deaths and no survivors were not prioritized in the humanitarian response and did not receive substantial food supplies or socioeconomic support from the NGO community. We identified these affected households without EVD survivors as food insecure in our study, serving as a reminder for the NGO/governmental community to consider them in future EVD support programs. For affected households with and without EVD survivors to achieve long-term economic stability, programs such as CPES should continue for a longer period and shift the focus from health and well-being to educational and employment opportunities over time as part of a long-term commitment to transition households and communities to sustainability and independence.

Our study has several limitations and strengths. This study was done in Kono District, where there is a higher proportion of famers and miners compared to other communities. Thus, it is difficult to generalize our findings to the general population of Sierra Leone or, indeed, of West Africa. However, these findings still highlight major concerns and themes on the socioeconomic impact of the Ebola in low-resource, rural settings. One of the strengths of this study was that in using an open-ended, semi structured interview format with probing, we were able to get a better glimpse of the complexity of food insecurity in EVD-affected households and minimize bias [55]. This allowed us to unravel a range of topics, including quality and quantity of food, short term and long-term humanitarian support, and physical health sequalae.

### Study implications

Our study describes the drivers of food insecurity and highlights the importance of timely, long-term comprehensive support in households impacted by Ebola during the 2013–2016 epidemic. As the COVID-19 pandemic persists and the frequency of Ebola epidemics continues to rise, it is imperative to continue to learn more about the devastating socioeconomic impact of outbreaks on households and create sustainable solutions and programs to address this pressing issue. Our goal is that the findings of this study will not only serve as a guide to the Sierra Leonean government and NGOs involved in epidemic response and relief, but it will also be used as a resource to other nations and communities that are dealing with Ebola or other disease outbreaks. We hope that this study will be a call to action for the humanitarian response to prioritize the socioeconomic stability of communities affected by EVD and other disease outbreaks to ensure people are able to meet the World Health Organization’s recommendation of a healthy diet [56].

## Supplementary Material

Inclusivity in Global Research

## Data Availability

The authors have made excerpts of the transcripts relevant to the study available within the paper. However, the raw data could not be publicly shared as they are open-ended interview that contains potentially identifiable and sensitive human participants’ information. Specific data request could be sent to Eugene, Richardson MD, PhD (Eugene_Richardson@hms.harvard.edu).
